# MG53 anchored by dysferlin to cell membrane reduces hepatocyte apoptosis which induced by ischaemia/reperfusion injury *in vivo* and *in vitro*

**DOI:** 10.1111/jcmm.13171

**Published:** 2017-04-12

**Authors:** Weifeng Yao, Haobo Li, Xue Han, Chaojin Chen, Yihan Zhang, Wai Lydia Tai, Zhengyuan Xia, Ziqing Hei

**Affiliations:** ^1^ Department of Anesthesiology Third Affiliated Hospital Sun Yat‐sen University Guangzhou Guangdong China; ^2^ State Key Laboratory of Pharmaceutical Biotechnology The University of Hong Kong Hong Kong China; ^3^ Department of Anaesthesiology The University of Hong Kong Hong Kong China; ^4^ Department of Anesthesiology Sun Yat‐sen Memorial Hospital Sun Yat‐sen University Guangzhou China

**Keywords:** hepatic ischaemia/reperfusion, mitsugumin‐53, dysferlin, reactive oxygen species

## Abstract

Hepatic ischaemia/reperfusion (HIR) induces severe damage on hepatocyte cell membrane, which leads to hepatocyte death and the subsequent HIR injury. In this study, we investigated the role and the mechanism of mitsugumin‐53 (MG53), a novel cell membrane repair protein, in protecting the liver against HIR injury. Rats were subjected to sham operation or 70% warm HIR with or without recombined MG53 (rhMG53), caudal vein‐injected 2 hrs before inducing HIR. *In vitro*, cultured hepatocyte AML12 cells were subjected to hypoxia/reoxygenation (H/R) in the presence of rhMG53 and/or dysferlin gene shRNAs or adenovirus transfection. HIR resulted in severe liver injury manifested as severe liver histological changes and increased AST and ALT release. Post‐ischaemic hepatic oxidative stress was significantly enhanced demonstrated by elevated dihydroethidium level, increased 4‐hydroxynonenal, enhanced 15‐F2t‐isoprostane and decreased SOD activity. rhMG53 administration attenuated post‐HIR liver injury, decreased liver oxidative stress and further enhanced dysferlin protein expression and its colocalization with MG53. Similarly, H/R induced AML12 cell injury and oxidative stress, which were abolished by either rhMG53 or dysferlin overexpression but were exacerbated by dysferlin gene knockdown. Dysferlin overexpression further increased H/R‐induced increased colocalization of MG53 and dysferlin. In conclusion, MG53 was anchored by dysferlin to reduce oxidative stress and cell death and attenuate HIR injury.

## Introduction

Ischaemia/reperfusion injury is a serious clinical complication in many organs including the liver, heart, brain and kidney 1. Hepatic ischaemia/reperfusion (HIR) injury mainly occurs during liver transplantation and hepatic resection 2. HIR by inducing overproduction of reactive oxygen species (ROS) and superoxide free radicals increases oxidative stress in the liver, which leads to the destruction of hepatic cell membrane and permeability, resulting in hepatocyte death and the subsequent HIR injury 3. Various strategies targeting certain stages of pathogenesis in HIR have been proven to alleviate the degree of injury in the liver 4. However, none of these strategies exerts protection by directly repairing cell membrane damage, which initiates cell/organ damage during HIR.

Mitsugumin‐53 (MG53), also known as TRIM72, is a member of the tripartite motif‐containing (TRIM) superfamily, which is constituted of proteins that possess a RING finger domain, one or two B‐boxes and a coiled‐coil domain 9. A series of studies identified MG53 as an essential component of the cell membrane repair machinery 5, 6, 7, 8. MG53 acts as a sensor of oxidative environment and will subsequently be oligomerized and recruit intracellular vesicles to sites of membrane disruption to form a repair patch 10. Genetic ablation of MG53 results in defective membrane repair, and MG53^−/−^ hearts are more susceptible to ischaemic reperfusion injury 11. Similarly, study has shown MG53 as a vital component of renal protection, and targeting MG53‐mediated repair of renal cells represents a potential approach to prevention and treatment of acute kidney injury 12. Recent study showed that MG53 transfection could significantly elevate MG53 protein expression in liver *in vitro* 13. However, the role of MG53 in HIR injury has not been investigated.

In order to confer its membrane repairing effect, MG53 needs to bind to a docking protein. Polymerase transcriptase release factor (PTRF) has been considered to be an important docking protein that binds to MG53 at the exposed membrane lipid at the injury site during MG53‐mediated membrane repair 14. However, PTRF is expressed in most organs but not in the liver 15. Dysferlin, another docking protein, involved in repair of plasma membrane damage, helps to recruit intracellular vesicles to site of damage in plasma membrane, appearing to have a ubiquitous distribution 16. Recently, study showed dysferlin deficiency is related to elevated aminotransferases which reflects the liver function 17. Also, the C2A domain in dysferlin has been reported to be important for association with MG53 18. All these provide a clue that dysferlin could anchor exogenous MG53 to the injury site to facilitate the recovery of damaged cells and repair of injured cell membranes during HIR. We, therefore, suggested that MG53 by binding to dysferlin attenuates HIR‐induced hepatocyte cell membrane damage, which decreases hepatocyte death and reduces oxidative stress, leading to the attenuation of HIR injury.

## Materials and methods

### Animals and hepatic warm ischaemia/reperfusion model

Male Sprague Dawley rats (aged 6–8 weeks, weighted 180–200 g, *n* = 8 per group) were obtained from SLAC Laboratory Animal Co. Ltd (Shanghai, China). Rats were raised and breed in Vaccine Institute Laboratory in the Third Affiliated Hospital of Sun Yat‐Sen University. After a midline laparotomy incision, an atraumatic vascular clip was placed on the vessels blocking the portal venous and hepatic arterial blood supply to the median and left lateral lobes of the liver, which resulted in approximately 70% rat liver ischaemia/reperfusion injury 19, 20. Animals in the sham groups were only received laparotomy and vessel separation without occlusion. Some of the rats received recombinant human MG53 protein (rhMG53, i.v., 5 mg/kg) or vehicle (0.9% sterile saline) 2 hrs 21 before surgery. The rhMG53 protein was obtained from Creative BioMart Company (Cat#. TRIM72‐156H). DNA sequence encoding the human MG53 (Gene ID: 493829) was fused with a polyhistidine tag at the C‐terminus. The rhMG53 protein was then expressed in *Escherichia coli* cells and purified with Ni‐affinity chromatography with a final purity >95% as determined by SDS‐PAGE. Animals were killed using carbon dioxide inhalation method and samples were collected after rats were subjected to partial liver ischaemia for 60 min and subsequent reperfusion for 6 or 24 hrs. All animal experiments were approved by the Animal Care and Use Committee of Sun Yat‐Sen University (Guangzhou, China) and followed the ‘Guide for the Care and Use of Laboratory Animals’ (NIH Publications no. 8023, revised 1978) guidelines for the treatment of animals.

### Liver injury assay

Histology analysis was performed as described in our previous study 22. Serum alanine aspartate aminotransferase (ALT) and aspartate aminotransferase (AST) levels were determined by a 7180 Biochemical Analyzer (Hitachi, Japan).

### Measurement of 15‐F2t‐isoprostane and superoxide dismutase activity

15‐F2t‐isoprostane (Cayman, USA) level in liver tissue homogenate was measured according to the manufacturer's instructions *via* enzyme‐linked immunosorbent (ELISA) assay. The activity of superoxide dismutase (SOD) was determined using a commercial kit (Nanjing Jiancheng Bioengineering Institute, Nanjing, China) as we described 22. SOD activity was expressed as unit/mg wet protein.

### Detection of apoptotic cell death by terminal deoxynucleotidyl transferase dUTP nick‐end labelling

Liver tissue section terminal deoxynucleotidyl transferase dUTP nick‐end labelling (TUNEL) reaction was detected using an *in situ* cell death detection kit (Roche Diagnostics GmbH, Mannheim, Germany). The section slides were observed under a light microscope by a person who was initially blinded to treatment groups, and ten randomly selected fields of each slide were chosen and analysed. Finally, apoptotic index was calculated as a percentage of apoptotic nuclei to total nuclei.

### Immunohistochemical assay

The 5‐μm liver paraffin‐embedded sections cut from the tissue blocks were subjected to standard procedure for dewaxing, blocking endogenous peroxidase and exposing antigenic sites before immunohistochemical staining. Mouse monoclonal IgG1 anti‐4HNE antibody (diluted at 1:500, Abcam, USA) was used as primary antibody. Positive signal was visualized with DAB (3,3‐diaminobenzidine Dako, Denmark) colour reaction. Nuclei were stained with haematoxylin. Under the code, five fields per each slide at random choice of the viewer were semi‐quantified.

### Immunofluorescence

The liver paraffin blocks were cut into 5‐μm sections; potential non‐specific staining in the sections was blocked with 5% bovine serum albumin and 0.1% Triton X‐100 in PBS. Mouse anti‐MG53 (1:200; Santa Cruz Biotechnology, Santa Cruz, CA, USA ) and dysferlin (1:500; Santa Cruz Biotechnology, Santa Cruz, CA, USA ) primary antibodies were used, followed by incubation with a secondary antibody conjugated with fluorescence (1:200; Life technologies, Carlsbad, CA, USA). Fluorescent microscope (Leica, DMLB2, Germany) was utilized for observing the stained sections. Ten randomly selected fields of each slide were chosen and analysed using the software ImageJ 1.48 (National Institutes of Health) according to its instructions.

### Immunoprecipitation and immunoblotting

Cultured hepatocytes or liver tissue was homogenized in lysis buffer. A total of 500 mg extracts was subjected to immunoprecipitation with 2 mg MG53 primary antibody or IgG as negative control in the presence of 20 ml protein A/G PLUS‐agarose. After extensive PBS washes, the immunoprecipitates were denatured with 13 sodium dodecyl sulphate loading buffer and subjected to analysis for DYSF and MG53 expression by Western blot as described below.

### Western blot analysis

The liver tissues were finely homogenized, suspended in ice‐cold lysis buffer and then centrifuged (12,000 *g*, 10 min) at 4°C. The homogenate supernatants were collected for subsequent analysis. After measuring the protein concentrations using bicinchoninic acid (BCA) method, a total 50 μg sample was solubilized in sodium dodecyl sulphate (SDS) loading buffer and loaded into a 10% SDS polyacrylamide gel, electrophoresed and then transferred to a polyvinylidene difluoride (PVDF) membranes. Finally, the PVDF membranes were incubated with dysferlin (1:1000; Santa Cruz Biotechnology), Bax, Bcl‐2, cytochrome *c*, caspase 3 and cleaved caspase 3 antibodies (1:1000, Cell Signaling Technology, Inc, USA), followed by the corresponding secondary antibodies. Protein–antibody complexes were detected with an enhanced chemiluminescence system (KGP1125, Nanjing KeyGEN Biotech. Co., Ltd.). Anti‐GAPDH (1:500, Santa Cruz Biotechnology) was used to detect protein levels. The density measurement was correlated to the protein levels and normalized to those of GAPDH.

### Cell culture and hypoxia/reoxygenation (H/R) model

Mice hepatocyte AML12 (ATCC) was maintained following the vendors’ recommendations. Hypoxia/reoxygenation (H/R) was performed as described in our previous study 23. In brief, cells were incubated for 24 hrs in Galaxy 48R hypoxia incubator (Eppendorf Company Hamburg, Germany) with hypoxia gas mixture (5% CO_2_, 94% N_2_ and 1% O_2_) at 37°C. And, following the completion of the corresponding hypoxia time, cells were then taken out from hypoxia incubator and transferred back to a regular incubator with 21% oxygen for 4 hrs. Cell survival was assessed using the Cell Counting Kit‐8 (CCK‐8) and lactate dehydrogenase (LDH) assays.

### rhMG53 delivery and DYSF shRNA and adenovirus transfection

The rhMG53 was respectively diluted with DMEM/F12 cell culture medium and added directly to the cells. After pre‐treatment for 24 hrs, the cells were transfected with or without DYSF short hairpin RNA (shRNA) plasmid (Santa Cruz Biotechnology, Inc) or DYSF‐adenovirus (Adv) (NM_001077694, GeneCopoeia, Inc, China) for 48 hrs and then H/R was induced as previously described 24. Recombinant adenovirus was generated by homologous recombination and amplified in HEK293 cells. Transient transfections were performed with Lipofectamine™ 3000 (Invitrogen, USA) according to the manufacturer's protocol as described in our previous study 24.

### Measurement of cellular reactive oxygen species (ROS)

Superoxide generation in liver tissue frozen section and cultured AML12 hepatocytes were estimated by the dihydroethidium (DHE) staining as described 25. Briefly, liver tissue frozen section or hepatocytes were loaded with DHE (10 μM, Sigma, USA) for 30 min at 37°C. The DHE fluorescence intensity was calculated in each of ten randomly selected fields and analysed as a percentage relative to control by a quantitative morphometric method.

### Mitochondrial membrane potential assay

As an early event in the mitochondrial apoptosis cascade, mitochondrial membrane potential (MMP) depolarization was measured using JC‐1 assay kit (Cayman, USA). Briefly, after experimental treatment, AML12 cells were cultured in 12‐well plates and incubated with JC‐1 staining solution (5 μg/ml) at 37°C for 15 min and washed twice with PBS. MMP was estimated by measuring the fluorescence ratio of free JC‐1 monomers (green) to JC‐1 aggregates in mitochondria (red) using fluorescence microscopy (Leica, DMLB2, Germany). Mitochondrial depolarization is indicated by an elevation in the ratio of hepatocyte emitting green fluorescence.

### Statistical analysis

Data are expressed as mean ± standard error of mean (SEM). Biochemical assays were performed in triplicate for each specific sample. Therefore, all the data points are means of numbers themselves resulting from means of triplicate measurements for these parameters. Significance was evaluated using *one‐way ANOVA* test (SPSS 13.0, SPSS Inc, Chicago, IL, USA) followed by *Tukey post hoc* multiple comparisons test for unpaired values. *P* < 0.05 was considered statistically significant.

## Results

### Liver MG53 is increased after HIR injury

**Figure 1:**
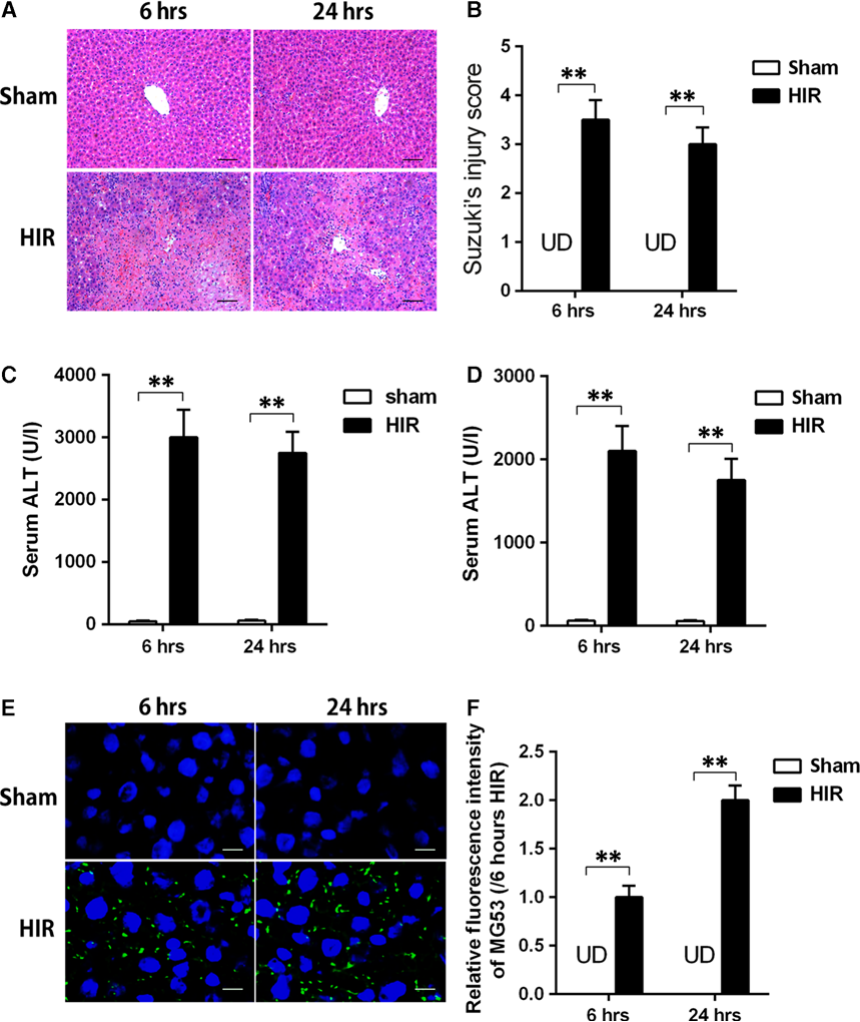
Liver MG53 expression was increased after hepatic ischaemia/reperfusion that was associated with severe liver injury and oxidative stress. Rats were subjected to HIR and samples were collected after liver reperfusion for 6 and 24 hrs. (**A**) Haematoxylin and eosin (H&E)‐stained sections of livers (×200) and Suzuki's scores; (**B**) serum aspartate aminotransferase (AST) level; (**C**) serum alanine aspartate aminotransferase (ALT) level; (**D** and **E**) immunofluorescence staining (×400) of liver MG53 (green) and nucleus (blue) and density analysis. Date are mean ± SEM, *n* = 8/group, **P < 0.01. HIR, hepatic ischaemia/reperfusion; UD, undetectable.

As shown in Figure 1, 6 and 24 hrs after reperfusion, there were severe histological alterations in the liver and significant increase of Suzuki's score (Fig. 1A and B). These changes were associated with increased AST and ALT (Fig. 1C and D), markers of hepatic injury in the HIR group, compared with the sham group. Interestingly, protein expression of MG53 was undetectable in the liver in the sham group (Fig. 1E and F), while hepatic MG53 protein expression was increased at 6 hrs after reperfusion and continuously elevated even at 24 hrs after reperfusion. As MG53 was recently considered as a repaired tool, which was called ‘woundplast’ protein during cell membrane damage, whether MG53 could repair the injured hepatocytes is still in need of investigation.

### MG53 supplementation improves hepatic function and ameliorates hepatocellular oxidative stress after HIR injury

Firstly, polyhistidine‐tagged MG53 (His‐rhMG53) protein was identified by SDS‐PAGE and Western blot. The results showed a good purity of His‐rhMG53 in different protein contents (0.1, 1, 2, 4 μg; Figure S1A). Furthermore, treatment with rhMG53 (0, 0.05, 0.10, 0.15 and 0.20 mg/ml) but not boiled rhMG53 robustly reduced LDH release which reflected cell membrane damage in a dose‐dependent manner in hepatocytes subjected to hypoxia 24 hrs/reoxygenation 4 hrs, suggesting that rhMG53 existed normal function against cell membrane damage (Figure S1B and C).

**Figure 2:**
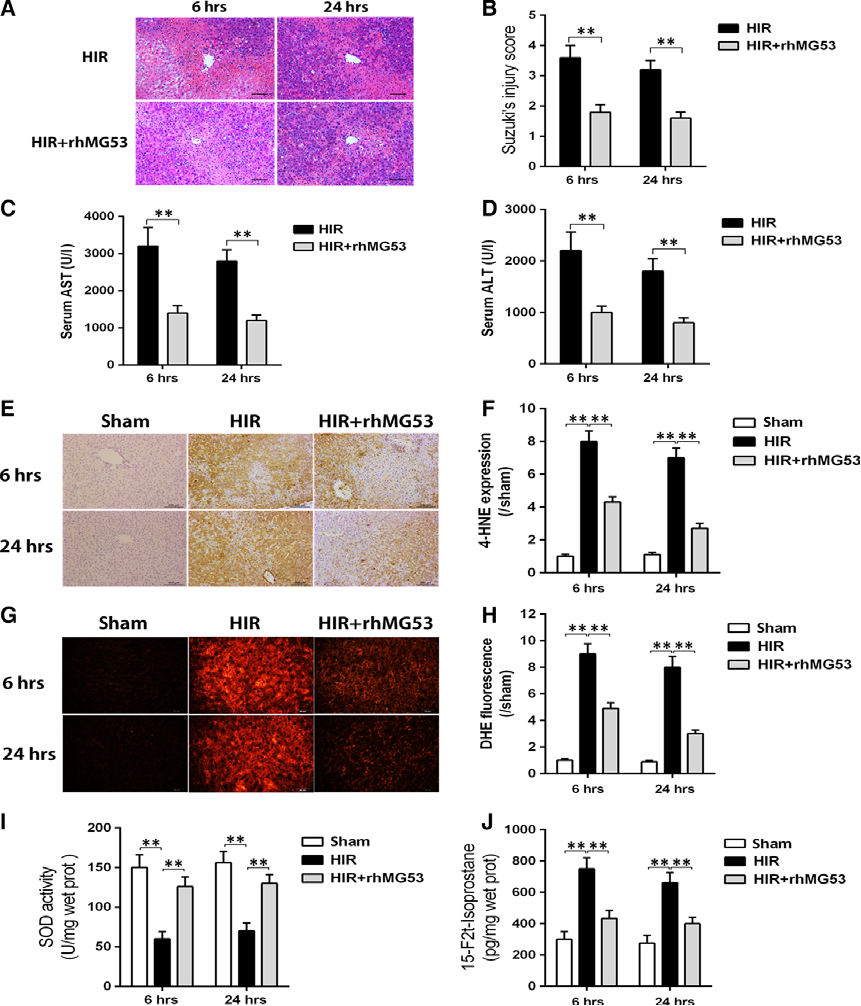
MG53 administration reduced hepatic injury and oxidative stress during ischaemia/reperfusion. Rats were subjected to HIR or sham operation with or without administration of rhMG53 *via* caudal vein and then samples were collected after liver reperfusion for 6 and 24 hrs. (**A** and **B**) Haematoxylin and eosin (H&E)‐stained sections of livers (×200) and Suzuki's scores; (**C**) serum aspartate aminotransferase (AST) level; (**D**) serum alanine aspartate aminotransferase (ALT) level; (**E** and **F**) reactive oxygen species (ROS) levels assessed using fluorescence microscopy following the changes in immunofluorescence staining (×200) of liver dihydroethidium (DHE) (red). (**G** and **H**) Immunohistochemical staining (×200) of liver 4‐hydroxynonenal (4‐HNE) and density analysis. (**I**) Liver antioxidant enzyme superoxide dismutase (SOD) activity; (**J**) liver oxidative stress marker 15‐F2t‐isoprostane level. Date are mean ± SEM, *n* = 8/group, **P < 0.01. HIR, hepatic ischaemia/reperfusion; rhMG53, recombinant human MG53 protein.

To test whether MG53 supplementation is effective in protecting against HIR‐induced liver injuries, a single dose of 5 mg recombined MG53 (rhMG53) protein/kg body weight was injected *via* caudal vein 2 hrs before the rat was subjected to HIR injury. Control rat received an equal volume of saline solution. As shown in Figure 2A and D, administration of rhMG53 prior to ischaemia could effectively reduce post‐ischaemic liver histological injury, accompanied with decrease in serum AST and ALT levels. These hepatoprotective effects of rhMG53 were observed at 6 and 24 hrs after reperfusion. Furthermore, generation of ROS and lipid peroxidation products was significantly increased in liver demonstrated by higher levels of DHE (Fig. 2E and F), 4‐hydroxynonenal (4‐HNE; Fig. 2G and H), 15‐F2t‐isoprostane (J) and decreased SOD activity (I) in the HIR group than those in the sham group at 6 and 24 hrs after reperfusion. All these changes were reversed by rhMG53 pre‐treatment.

### MG53 administration prevents HIR injury through alleviating hepatocyte apoptosis

**Figure 3:**
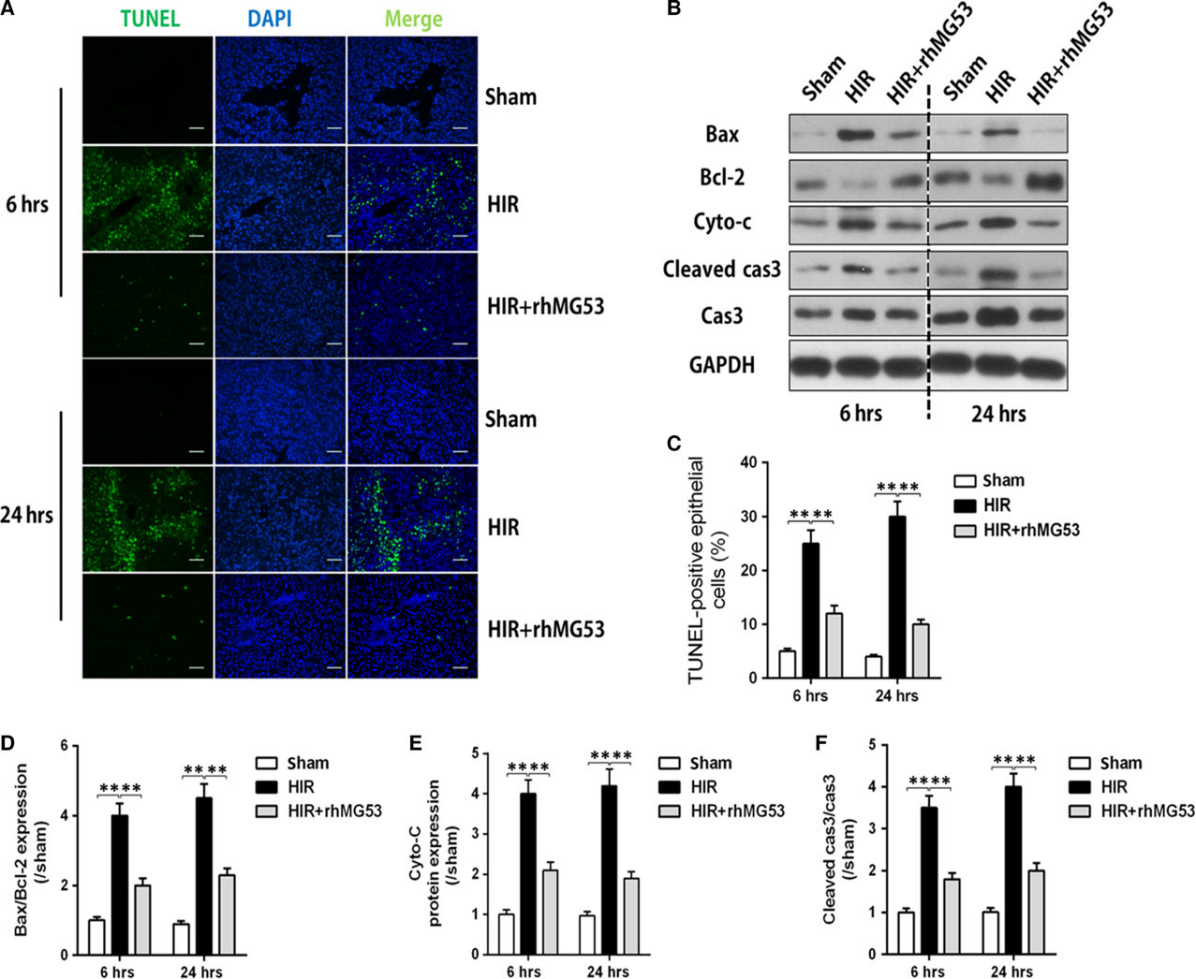
Protective effects of rhMG53 were related to hepatic apoptosis inhibition. Rats were subjected to HIR or sham operation with or without administration of rhMG53 *via* caudal vein and then samples were collected after liver reperfusion for 6 and 24 hrs. (**A**) Terminal deoxynucleotidyl transferase‐mediated nick‐end labelling (TUNEL) staining (×200) of liver; (**B**) Bax/Bcl‐2, caspase 3/cleaved caspase 3 and cytochrome *c* (Cyto‐*c*) protein expression; (**C**) percentage of TUNEL‐positive cells; (**D** and **F**) density analysis of Bax/Bcl‐2, caspase 3/cleaved caspase 3 and cytochrome *c* (Cyto‐*c*) protein expression. Date are mean ± SEM, *n* = 8/group, **P < 0.01. HIR, hepatic ischaemia/reperfusion; rhMG53, recombinant human MG53 protein.

As shown in Figure 3, post‐ischaemic hepatocyte apoptosis was significantly increased and manifested by the upsurge of TUNEL‐positive cells (Fig. 3A and C), Bax to Bcl‐2 ratio, cytochrome *c* protein expression and cleaved caspase 3 expression (Fig. 3B and D–F). All these changes were prevented/reversed by rhMG53 pre‐treatment (Fig. 3). Together, these results suggest that the reduction of apoptotic cell death contributes to MG53‐mediated hepatic protection following HIR injury.

### MG53 attenuates HIR injury *via* interaction with dysferlin

**Figure 4:**
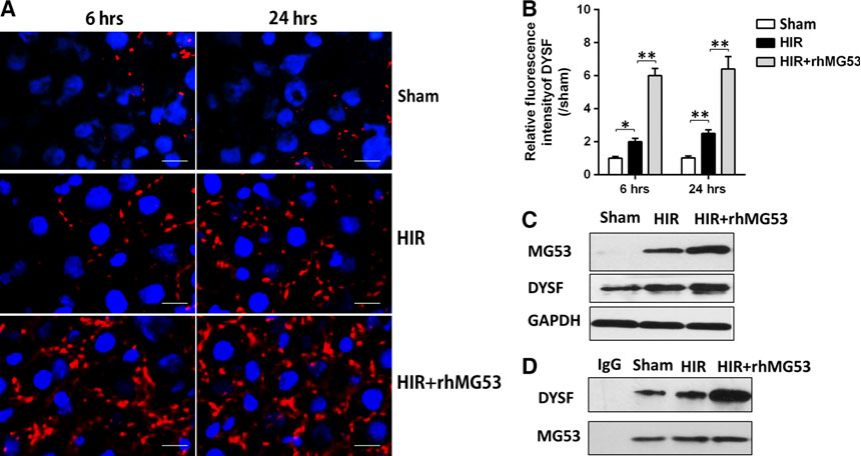
rhMG53 attenuated hepatic ischaemia/reperfusion injury *via* interaction with dysferlin. Rats were subjected to HIR or sham operation with or without administration of rhMG53 *via* caudal vein and then samples were collected after liver reperfusion for 6 and 24 hrs. (**A** and **B**) Immunofluorescence staining (×400) of liver dysferlin (DYSF) (Red) and nucleus (blue) and density analysis; (**C**) Western blot assay on liver dysferlin and MG53 at 6 hrs after reperfusion. (**D**) Immunoprecipitation assay of dysferlin/MG53 at 6 hrs after reperfusion. Date are mean ± SEM, *n* = 8/group, *P < 0.05, **P < 0.01. HIR, hepatic ischaemia/reperfusion; rhMG53, recombinant human MG53 protein.

We then investigated the role of dysferlin, a membrane protein that has been considered to interact with MG53, in MG53‐mediated hepatoprotection. As shown in Figure 4A–C, hepatic dysferlin expression was markedly increased in the HIR group compared with that in the sham group at 6 and 24 hrs after reperfusion. As shown in Figure 4D, co‐immunoprecipitation results showed that, in the sham group at 6 hrs after reperfusion, there was no colocalization of MG53 and dysferlin, while after HIR, colocalization of MG53 and dysferlin was significantly increased. This colocalization of MG53 and dysferlin was further enhanced after rhMG53 pre‐treatment. These results indicated that dysferlin may take an important part in rhMG53‐mediated protection against HIR injury.

### Down‐regulation of dysferlin reversed the protective effects of MG53 on hepatocyte H/R damage

**Figure 5:**
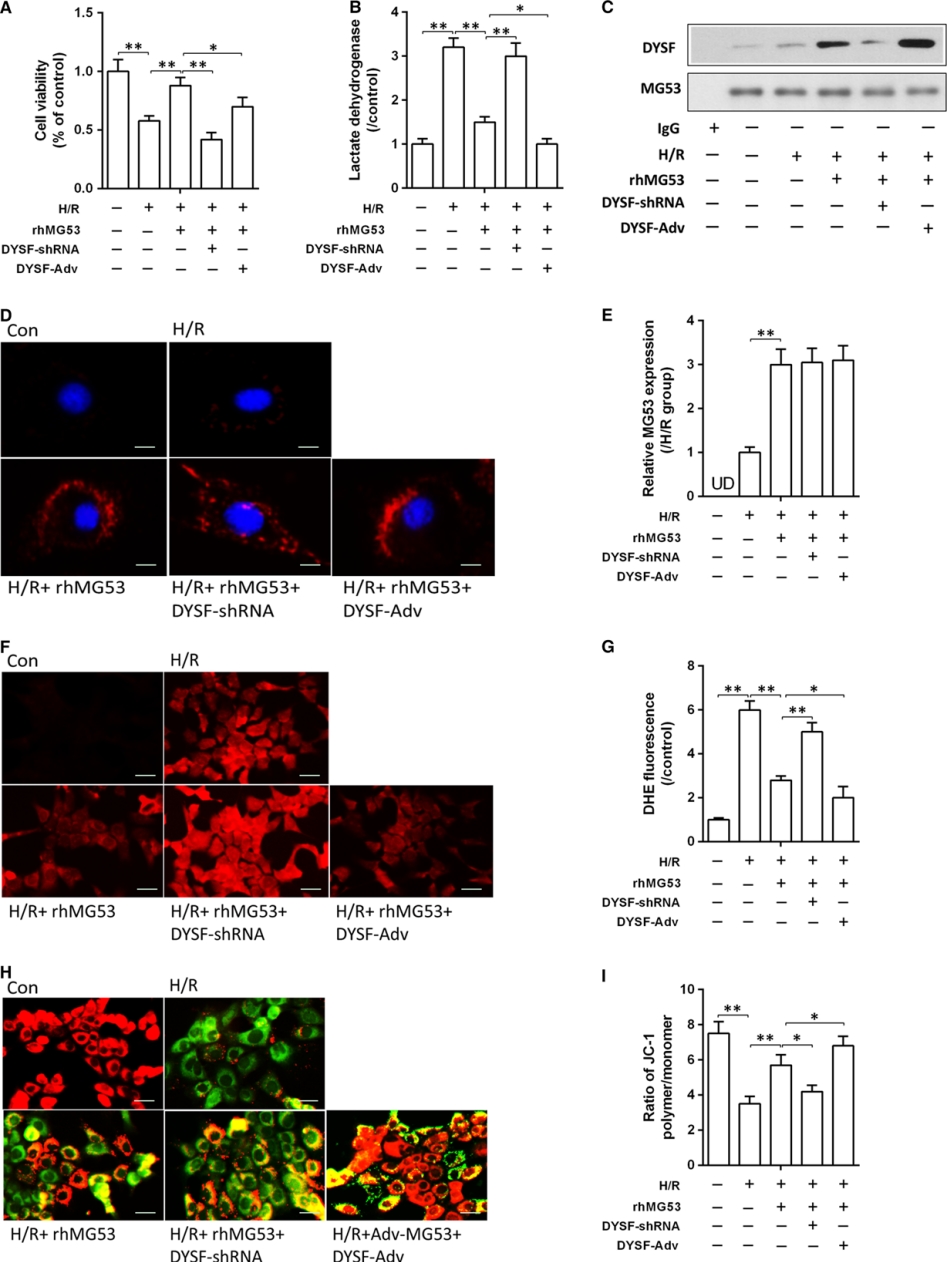
Down‐regulation of dysferlin reversed the protective effects of rhMG53 on hepatocyte H/R damage. (**A** and **B**) Cell proliferation was detected by CCK‐8 and cellular injury analysed by measuring LDH release in AML‐12 hepatocytes after H/R stimulation; (**C**) immunoprecipitation assay of dysferlin/MG53 of hepatocytes. (**D**) Immunofluorescence of MG53 protein expression on single hepatocyte using laser confocal microscopy (×1000); (**E**) density analysis of MG53 protein expression; (**F** and **G**) reactive oxygen species (ROS) levels assessed using fluorescence microscopy following the changes in DHE fluorescence (×400); (**H** and **I**) mitochondrial membrane potential (MMP) loss measured using a JC‐1 mitochondrial membrane potential assay method (×400). Date are mean ± SEM, and each experiment was performed at least independently in triplicate, *P < 0.05, **P < 0.01. H/R, hypoxia/reoxygenation; rhMG53, recombinant human MG53 protein; DYSF, dysferlin; UD, undetectable.

In order to confirm the role of MG53 in protecting the liver against HIR injury and its interplay with dysferlin, hepatocyte AML‐12 cells were subjected to H/R stimulation with or without rhMG53 in the presence or absence of dysferlin gene knockdown or overexpression by corresponding shRNAs or adenovirus transfection. As shown in Figure 5, stimulation with H/R significantly induced cell injury manifested as reduced cell viability, increased LDH release and reduced mitochondrial potential (decrease of JC‐1 polymer/monomer ratio; Fig. 5A and B, H and I), accompanied with increased oxidative stress demonstrated by increased intensity of DHE fluorescence (*P* < 0.01 *versus* control; Fig. 5F and G). All these changes were abolished or prevented by either rhMG53 supplementation or DYSF gene overexpression (*P* < 0.01 *versus* H/R group), while the beneficial effects of rhMG53 were abolished by DYSF gene knockdown. rhMG53 administration significantly enhanced MG53 cell membrane accumulation (*P* < 0.01 *versus* H/R group; Fig. 5D and E), accompanied with increased colocalization of DYSF and MG53, which was further increased by DYSF overexpression with Adv‐DYSF, but was reduced by DYSF gene knockdown (*P* < 0.05 *versus* H/R + rhMG53 group; Fig. 5C).

## Discussion

In current study, we demonstrated that MG53 supplementation, by interacting with dysferlin, reduced hepatic oxidative stress and hepatocyte death and attenuated hepatic ischaemia/reperfusion (HIR) injury. We showed that post‐ischaemic hepatic oxidative stress and hepatocyte death were significantly increased, which was associated with enhanced MG53 and dysferlin protein expression and their colocalization. We further demonstrated that supplementation of MG53, by increasing dysferlin and its interaction, reduced hepatic oxidative stress and attenuated hepatocyte death that eventually attenuated HIR injury. These findings were confirmed in our *in vitro* model which showed that supplementation of MG53 or dysferlin overexpression attenuated hypoxia/reoxygenation injury in cultured AML12 cells. In contrast, these protective effects of MG53 or dysferlin were cancelled by dysferlin gene knockdown, indicating the importance of MG53/dysferlin in HIR injury. To our knowledge, this is the first study investigating the protective role of MG53 in HIR injury.

The liver is sensitive to ischaemia/anoxia injury 26, 27. Ischaemia/reperfusion causes acute liver ischaemia/anoxia and subsequently a series of significant hepatic morphological changes 28. The hepatocytes are vulnerable to ischaemia/anoxia damage 29. Experimental evidence suggests that HIR injury is biphasic: early (2–6 hrs after reperfusion) ischaemia/reperfusion (I/R) injury occurs with the initiation of an inflammatory cascade involving the formation of numerous ROS and reactive nitrogen species, and the release of chemokines and cytokines, followed by neutrophil‐mediated oxidative stress injury occurring at 6–24 hrs of reperfusion. Oxidative stress further damages the cell membrane and finally causes irreversible hepatocyte injury or death between the mitochondrial inner and outer membranes 30. Release of cytochrome *c* occurs in the situation when large amplitude mitochondrial swelling following the mitochondrial permeability transition causes rupture of the outer mitochondrial membrane 31. The change of pro‐apoptotic Bcl‐2 family members such as Bax could also cause the release of cytochrome *c* and other pro‐apoptotic mitochondrial factors during cell apoptosis 32. In HIR injury, the oxidative injury to the enzyme complexes and reduction of anti‐apoptotic protein triggered apoptosis 33. Hoglen *et al*. 34 found that inhibition of caspases markedly attenuated liver damage after HIR which suggested that apoptosis plays a critical role in HIR injury. The apoptosis of indigenous hepatocytes leads to a generation of AST and ALT, further demoting liver function 35. In our rat model, after exogenous rhMG53 administration, we observed a significant increase of MG53 protein expression in the liver, indicating that exogenous supplementation of MG53 is sufficient to gain liver MG53 level. Furthermore, we showed that exogenous rhMG53 administration significantly alleviated post‐HIR liver damage that was associated with reduced hepatic oxidative stress and hepatocyte death, suggesting that MG53‐reduced HIR injury may act by decreasing oxidative stress and cell death. We also detected the hepatic SOD activity and reactive oxygen species generation which reflected oxidative stress derived from mitochondria. More interestingly, we found that rhMG53 treatment could also decease hepatic oxidative stress derived from mitochondria, demonstrated as enhancement of SOD and reduction of ROS. This finding indicates that MG53 may also play a guardian role in mitochondrial membrane protection. These results were consistent with the those of the previous study by Ma *et al*. 36 which showed subcellular MG53 could also localize around mitochondria. Nonetheless, how MG53 reduced HIR‐induced oxidative stress and hepatocyte death is still unclear.

MG53 is one of the unique muscle TRIM family proteins, and it is previously thought to be expressed only in cardiac/skeletal muscles 37. However, recent study also showed MG53 played a role of damage repair in other organ such as liver, kidney and the cortex 12, 13. In the current study, we found that under physiological condition, MG53 cannot be detected in the liver, while under pathological condition such as HIR, MG53 is expressed in the liver, and our results provided additional evidence that MG53 is indeed expressed in the liver and its expression may be inducible by stress stimulus.

MG53 was showed to modulate caveolar endocytosis in repair of lung cells 38, 39. It is an essential component for cell membrane injury with the repair mechanism. After membrane injury, MG53 receives the initial signal from membrane injury such as the exposure of membrane lipid molecules 40. Mediated by docking protein, MG53 is then transferred to the injured membrane in cellular vesicles and bounds to the membrane lipid molecules which were exposed during cell damage. Subsequently, the vesicles could merge with the cytoplasmic membrane to initiate the repair process 10. As also shown by previous data, acute membrane damage signals could be detected by environmental MG53 proteins, which then recruited and repaired the damaged cell membrane. However, MG53 could not bind to the exposed membrane lipid molecules by itself; thus, another mediating molecule is required at the injury site to anchor MG53 to the exposed membrane lipid molecules 11. Dysferlin, bands at ~230 kDa, appears to have a ubiquitous distribution and is observed in all organ tissues tested. Skeletal muscle, kidney and heart showed the strongest expression level; lung, stomach, spleen and liver and uterus also showed clear labelling, with nervous tissue including spinal cord, brain stem, cerebellum and sciatic nerve showing less 41. We used immunoprecipitation assays to investigate the correlation between MG53 and the dysferlin protein, and we found that MG53 is associated with dysferlin during HIR injury. HIR injury promoted dysferlin accumulation at damaged hepatocyes, and *in vivo* experiment showed MG53 trafficking to the damaged site requires dysferlin. Based on immunohistochemistry, we noted that exogenous rhMG53 was distributed in rat hepatocytes, and the histopathological characteristics of the tissue were significantly improved compared with the control group. Based on our immunohistochemistry results, we noted numerous dysferlin‐positive cells in the liver of the injured group and there was no distinct difference between the injured and control groups. Exogenous rhMG53 protein's distribution in the liver was consistent with the expression of dysferlin expression. All of the above results indicate that the selective distribution and repair mediated by exogenous MG53 correlates closely with dysferlin in the liver during HIR injury.

Of note, current study proved that the cell membrane repair by MG53 accumulation in hepatocytes during HIR is mediated by dysferlin, which reduces oxidative stress and attenuates hepatocyte death in HIR. We could not exclude the possibility that MG53 may have a direct effect on antioxidant enzyme or anti‐apoptosis factors, which remains unknown. Moreover, whether there are other specific proteins that could recruit MG53 to bind to hepatocyte cell membrane rather than dysferlin also needs to be investigated in the future.

In conclusion, for the first time, we showed that exogenous MG53 can effectively interact with dysferlin, and thereby ameliorates hepatic oxidative stress, attenuates hepatocyte death and eventually attenuates severe hepatic ischaemia/reperfusion injury. Our data provide a novel mechanism for protecting the liver from injury that may be important for future treatment of hepatic ischaemia/reperfusion injury in the clinic.

## Conflict of interest

The authors declare no competing interests.

## Author contributions

**Figure 6:**
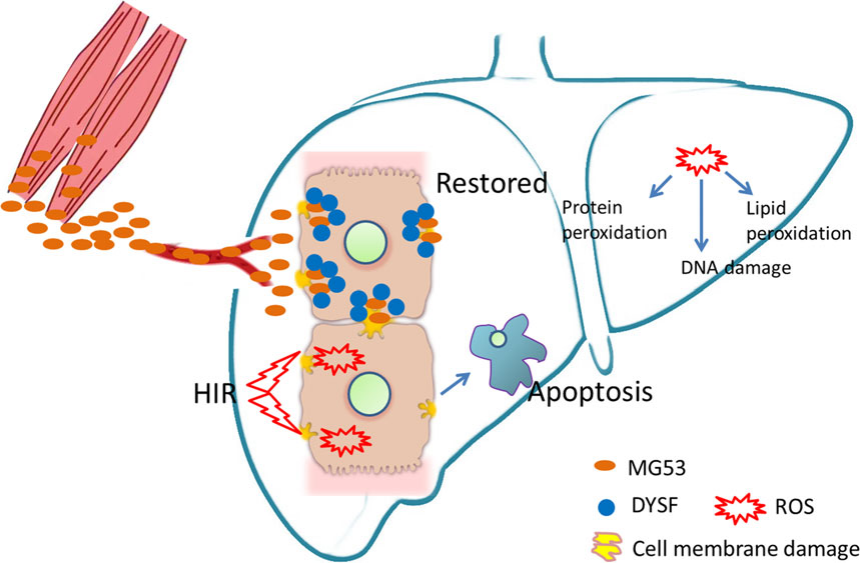
Schematic of proposed signalling involved in protective effects of MG53 on HIR injury. Hepatocyte cell membrane was damaged by ischaemia/hypoxia and aggravated by reperfusion/reoxygenation. Apoptosis is a main cell death pathway of hepatocytes subjected to ischaemia/reperfusion or hypoxia/reoxygenation. MG53 could promote repair of damaged cell membrane anchored by dysferlin, accompanied with decrease of hepatocytes oxidative stress which was manifested by ROS generation‐caused lipid peroxidation, DNA damage and protein peroxidation.

Ziqing Hei and Weifeng Yao conceived and designed the experiments. Weifeng Yao, Haobo Li, Xue Han, Chaojin Chen, Yihan Zhang and Wai Lydia Tai performed the experiments. Weifeng Yao and Haobo Li analysed the data. Zhengyuan Xia contributed reagents/materials/analysis tools. Weifeng Yao wrote the manuscript. Figure 6 image was drawn by Weifeng Yao. All authors read and approved the manuscript.

## Supporting information


**Figure S1** Identify of the rhMG53 protein and its functional test against cell membrane damage. **A**, SDS‐PAGE and western blot were carried out to show the purity and identify of the His‐MG53 protein in different protein contents. **B**, The functional test of rhMG53 was evaluated by using H/R‐induced AML12 hepatocytes. Different hypoxia/reoxygenation (H/R) time course (H4R4, H8R4, H12R4, and H24R4) were carried out, and His‐MG53 (0.20 mg/ml) was pretreated. **C**, The treatment with rhMG53 (0, 0.05, 0.10, 0.15, and 0.20 mg/ml) but not denatured rhMG53 (boiled for 20 min) robustly reduced LDH release which reflected cell membrane damage in a dose‐dependent manner in hepatocytes subjected to H/R (hypoxia 24 hours/reoxygenation 4 hours). Date are mean ± SEM, each experiment was performed at least independently in triplicate, *P < 0.05, **P < 0.01. rhMG53/His‐MG53: recombinant human MG53 protein.
