# Supplementary figures and images for: MG53 anchored by dysferlin to cell membrane reduces hepatocyte apoptosis which induced by ischaemia/reperfusion injury in vivo and in vitro
Source: J Cell Mol Med. 2017 Apr 12;21(10):2503–13. doi: 10.1111/jcmm.13171 (PMC5618678; doi:10.1111/jcmm.13171)

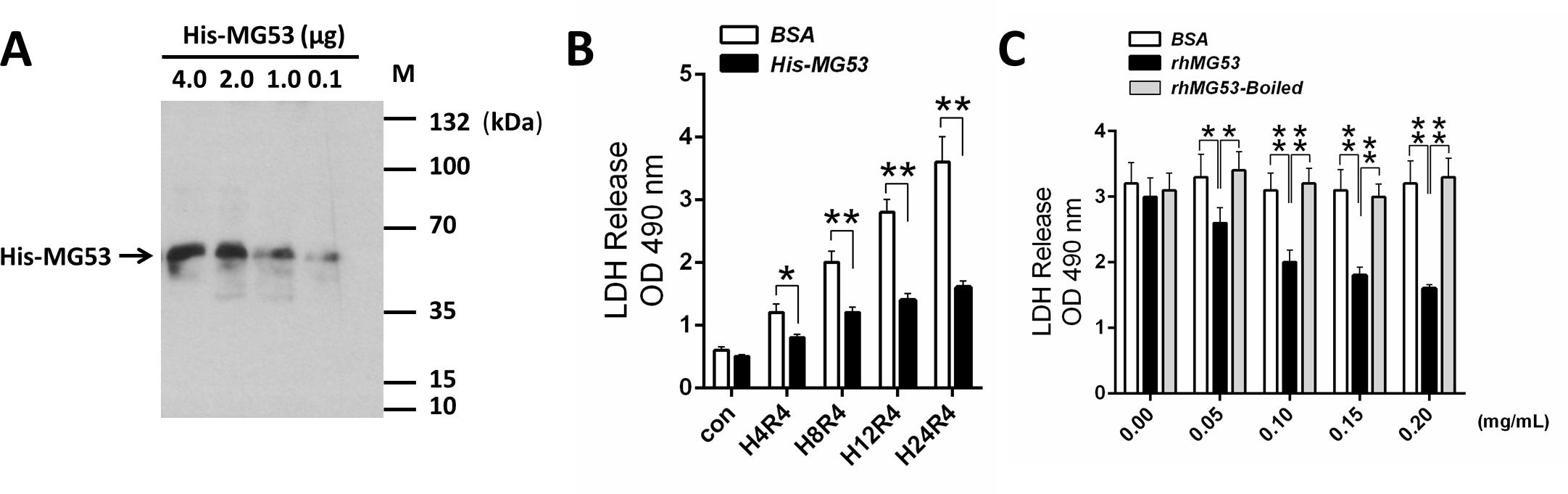

Supplement: Supplementary file 1 — Figure S1 Identify of the rhMG53 protein and its functional test against cell membrane damage. A, SDS‐PAGE and western blot were carried out to show the purity and identify of the His‐MG53 protein in different protein contents. B, The functional test of rhMG53 was evaluated by using H/R‐induced AML12 hepatocytes. Different hypoxia/reoxygenation (H/R) time course (H4R4, H8R4, H12R4, and H24R4) were carried out, and His‐MG53 (0.20 mg/ml) was pretreated. C, The treatment with rhMG53 (0, 0.05, 0.10, 0.15, and 0.20 mg/ml) but not denatured rhMG53 (boiled for 20 min) robustly reduced LDH release which reflected cell membrane damage in a dose‐dependent manner in hepatocytes subjected to H/R (hypoxia 24 hours/reoxygenation 4 hours). Date are mean ± SEM, each experiment was performed at least independently in triplicate, *P < 0.05, **P < 0.01. rhMG53/His‐MG53: recombinant human MG53 protein. [file JCMM-21-2503-s001.tif]
